# Salvianolic acid B functioned as a competitive inhibitor of matrix metalloproteinase-9 and efficiently prevented cardiac remodeling

**DOI:** 10.1186/1471-2210-10-10

**Published:** 2010-08-25

**Authors:** Baohong Jiang, Jing Chen, Lingling Xu, Zhenting Gao, Yanping Deng, Yanhui Wang, Feng Xu, Xu Shen, De-an Guo

**Affiliations:** 1 Shanghai Institute of Materia Medica, Chinese Academy of Sciences, Shanghai 201203, China; 2 School of Pharmacy, East China University of Science and Technology, Shanghai 200237, China; 3 Shenyang Pharmaceutical University, Wenhua Road #103, Shenyang 110016, China

## Abstract

**Background:**

Infarct-induced left ventricular (LV) remodeling is a deleterious consequence after acute myocardial infarction (MI) which may further advance to congestive heart failure. Therefore, new therapeutic strategies to attenuate the effects of LV remodeling are urgently needed. Salvianolic acid B (SalB) from *Salviae mitiorrhizae*, which has been widely used in China for the treatment of cardiovascular diseases, is a potential candidate for therapeutic intervention of LV remodeling targeting matrix metalloproteinase-9 (MMP-9).

**Results:**

Molecular modeling and LIGPLOT analysis revealed *in silico *docking of SalB at the catalytic site of MMP-9. Following this lead, we expressed truncated MMP-9 which contains only the catalytic domain, and used this active protein for in-gel gelatin zymography, enzymatic analysis, and SalB binding by Biacore. Data generated from these assays indicated that SalB functioned as a competitive inhibitor of MMP-9. In our rat model for cardiac remodeling, western blot, echocardiography, hemodynamic measurement and histopathological detection were used to detect the effects and mechanism of SalB on cardio-protection. Our results showed that in MI rat, SalB selectively inhibited MMP-9 activities without affecting MMP-9 expression while no effect of SalB was seen on MMP-2. Moreover, SalB treatment in MI rat could efficiently increase left ventricle wall thickness, improve heart contractility, and decrease heart fibrosis.

**Conclusions:**

As a competitive inhibitor of MMP-9, SalB presents significant effects on preventing LV structural damage and preserving cardiac function. Further studies to develop SalB and its analogues for their potential for cardioprotection in clinic are warranted.

## Background

Due to therapeutic interventions that limit infarct size and restore blood flow, short-term survival following a myocardial infarction (MI) has greatly improved [1]. The consequence of MI in patients is the left ventricular (LV) remodeling which leads to congestive heart failure [2]. LV remodeling accompanied with changes in heart wall structure, chamber geometry, and pump function is coordinated through the synthesis and degradation of extracellular matrix (ECM) [3]. ECM turnover is tightly regulated, particularly through the matrix metalloproteinases (MMP), a family of proteolytic enzymes [4]. Throughout the LV remodeling process after MI, MMPs release from inflammatory and endogenous cells, degrade ECM, disengage integrins, and stimulate reparative fibrosis [5]. Therefore, developing specific MMP inhibitors is an important therapeutic strategy against post-MI remodeling. Earlier studies have shown that MMP-9 is prominently over-expressed in the early phase following MI which correlates with functional deterioration of heart [6]. In addition, animals with MMP-9 genetic deletion or deficiency have shown improved preservation of cardiac function post-MI, suggesting that MMP-9 is a potential target for cardiovascular drug development [7-10].

*Salviae Miltiorrhizae*, one of the most important traditional herbal medicines, is widely used in clinic in China, Japan, and other countries for the treatment of coronary artery disease and other cardiovascular diseases [11]. SalB, the most abundant and bioactive ingredient of *Salviae Miltiorrhizae*, has raised a considerable interest in recent years. It has been assigned as the marker component of *Salviae Miltiorrhizae *in the Chinese Pharmacopoeia. Our recent studies have shown that salvianolic acids, which contains 65% of SalB, could significantly inhibit MMP-9 but not MMP-2 activity at infarct myocardium of rat with MI [12]. However, the molecular target of SalB has yet to be defined although multiple pharmacological effects of SalB have reported.

In the current study, we demonstrated the direct binding of SalB to MMP-9 and association of the cardio-protection of SalB with its competitive inhibitory effect on MMP-9 activity. These data will not only provide molecular basis for SalB as MMP-9 inhibitor, but also support to further develop more selective MMP-9 inhibitors against cardiac remodeling.

## Methods

### Animal model

Wistar male rats (230-250 g) were purchased from Shanghai Center of Experimental Animals, Chinese Academy of Sciences. MI was introduced by ligating the left anterior descending coronary artery near the main pulmonary artery as described previously [12] Rats were randomly assigned into four groups: sham operated rats given saline (Sham, n = 30) or SalB (Sham-SalB, n = 20); MI rats given saline (MI, n = 20) or SalB (MI-SalB, n = 20). After the surgery, saline or SalB (10 mg/kg) was administered by daily intravenous injection for 2 weeks. Animals were euthanized at 8 weeks after infarction. The hearts were harvest after arresting with a cold hyperkalemic solution. The cardioprotective effects of SalB were evaluated by echocardiography, hemodynamic parameters, and histological stain. To detect the protein expression level and activity of MMP-9 after MI, MI rats (n = 10) were sacrificed at 24 h after MI. "Guide for the Care and Use of Laboratory Animals" published by the US National Institutes of Health was followed throughout the study.

### Molecular Modeling

The three-dimensional structure of SalB was constructed using Corina online demonstration http://www.molecular-networks.com/online_demos/corina_demo. The model of MMP-9 protein with complete sequence was retrieved from the Protein Data Bank (PDB) at the Research Collaboratory for Structural Bioinformatics http://www.RCSB.org. The PDB entry 2OVX was selected for the three-dimensional structural model of MMP-9, because it was an MMP-9/inhibitor complex with the highest resolution among all available MMP-9 complex structures [13]. The docking of SalB to MMP-9 was calculated using version 3.0.5 of AutoDock.

### Construction of catalytic domain of MMP-9

The full-length MMP-9 cDNA (MGC-12688) was purchased from American Type Culture Collection. The truncated catalytic domain of MMP-9 (MMP-9 CD) comprising residues 107-216 and 391-444 was constructed and cloned into a pET-15b vector (Novagen), resulting in expression plasmid pET15b-MMP-9 CD. The recombinant protein MMP-9 CD was expressed in *E. coli*. BL21 (DE3) strain using pET15b-MMP-9 CD. The inclusion bodies were dissolved in 50 mM Tris-HCl (pH 7.5), 10 mM CaCl_2_, 500 mM NaCl, 8 M urea, and the protein was refolded by dialysis against urea free buffer (50 mM Tris-HCl (pH 7.5), 10 mM CaCl_2_, 500 mM NaCl, 20 μM ZnCl_2_, 10% glycerol) and finally against the same buffer in which 20 μM ZnCl_2 _and 10% glycerol were omitted. MMP-9 CD was then purified to homogeneity on zinc chelate chromatography column.

### In-gel gelatin zymography

The enzymatic activities of MMP-9 CD or tissue samples were detected by in-gel gelatin zymography [12]. Tissue samples were detected at 24 h after infarction. In brief, twenty μg protein from tissue or corresponding amount of protein for MMP-9 CD was electrophoresed in 10% SDS-PAGE under non-reducing conditions, containing 1% gelatin as MMP substrate. After electrophoresis, the gel was rinsed in 1% Triton X-100 for 1 h, washed in water, and incubated overnight in substrate buffer containing 50 mM Tris-HCl, 5 mM CaCl_2_, and 150 mM NaCl (pH 7.5) at 37°C with gentle shaking. Then, the gel was stained in 0.1% Coomassie Blue R-250 and destained using 10% methanol, 5% acetic acid solution. The protein species corresponding to MMP-9 were quantified using a MiniBis system (DNR Bio-Imaging Systems Ltd), and molecular weights were estimated using Prestained SDS-PAGE standards (Hou-Bio Tech. Ltd).

### Real time binding measured by surface plasmon resonance

The binding studies were performed using Biacore 3000 instrument (Biacore AB). All experiments were carried out using HBS-EP (10 mM HEPES pH 7.4, 150 mM NaCl, 3.4 mM EDTA and 0.005% surfactant P20) as running buffer with a constant flow rate of 30 μL/min at 25°C. MMP-9 CD protein, 5.8 μM in 10 mM sodium acetate buffer (pH 3.73), was covalently immobilized onto the CM5 sensor chip (BIAcore AB) using standard primary amine coupling procedure. SalB was dissolved in the running buffer with different concentrations ranging from 0 to 70 μM. The data was analyzed by BIAevaluation software, and the sensorgrams were processed by automatic correction for nonspecific bulk refractive index effects.

### The mode of inhibition by SalB on MMP-9

The inhibitory effect of SalB on hydrolysis of thiopeptolide (Ac-Pro-Leu-Gly-S-Leu-Leu-Gly-OEt) by MMP-9 CD was determined as previously described with modifications [14]. Briefly, SalB at indicated concentrations was incubated with MMP-9 CD (15 nM) in the reaction mixtures containing 1 mM 5,5'-dithiobis-(2-nitrobenzoic acid) at 4°C for 30 min. The reaction was then initiated by adding thiopeptolide to the reaction mixture, and monitored at 412 nm using Benchmark Plus™microplate spectrophotometer (Bio-Rad) at room temperature. Kinetic analysis of SalB against MMP-9 CD was calculated using double reciprocal plots of 1/V versus 1/[thiopeptolide]. The slope of every double reciprocal plot is the K_m_^app ^of enzyme at different SalB concentration. Secondary plot was drawn through K_m_^app ^versus SalB concentration. K_m_^app ^is apparent value of K_m_; K_i _is inhibition constant; K_i _was calculated using the equation Ki=[K_m_][SalB]/(K_m_^app ^-K_m_).

### Western blot for MMP-9

MMP-9 protein expression was detected at 24 h after infarction. Total protein from the infarct area was extracted and quantified. The aliquots of 20 μg protein were subjected to SDS-PAGE and the proteins were transferred to nitrocellulose transfer membrane. The membranes were then incubated with MMP-9 antibody at a 1:1000 dilution. The bands of MMP-9 were visualized by the chemiluminescence reagent and the density of the band was quantified. To validate that same amount of proteins were loaded on each lane; the membrane was stained with amino black.

### Functional assessment by echocardiography

Transthoracic echocardiography was performed using a commercially available echocardiographic Vevo 770 high-resolution ultrasound scanner (VisualSonics Inc., Toronto, Ontario, Canada) equipped with an RMV716 linear-array transducer for rat. M-mode tracings were used to measure LV wall thickness. Measurements were averaged from ten different readings per rat.

### Measurements of hemodynamic parameters

Eight weeks after surgery, the rats were anesthetized with ketamine (100 mg/kg). A Mikro-tipped SPR-320 catheter (Millar Instruments Inc) was inserted through the right carotid artery into left ventricle. Heart rate, mean arterial pressure (MAP), left ventricular systolic pressure (LVSP), end-diastolic pressure (EDP) of rats from different treatment groups were recorded using PowerLab 8/30 instrument (ADInstruments). Maximum rate of pressure development (+dP/dt_max_) and maximum rate of relaxation (-dP/dt_min_) were all derived or calculated from the continuously obtained pressure signal. All the parameters were analyzed using Chart 5 Pro software (ADInstruments).

### Measurement of cardiac output

A TS420 flowmeter and a transonic perivascular MA2.5 PSL flow probe (Transonic Systems, NY, USA), suitable for vessels of 0.7-1.2 mm OD, were used. The low-pass filter was set at 160 Hz. Data was acquired using a PowerLab recording unit at 1000 Hz. Briefly, rats were anaesthetized with ketamine (100 mg/kg), placed on a heating pad and ventilated. An upper two-thirds median sternotomy was performed. The thymus lobes were pulled apart to expose the aorta. The ascending aorta was dissected and the probe was then positioned around it. A micro-manipulator was used to carry the weight of the probe. An appropriate amount of ultrasound transmission gel was injected through the acoustic window of the probe to fill the air space between the probe and the aorta. Flow signals were found to be satisfactory throughout the preparation.

### Histopathological detection

The heart samples were fixed with 4% neutral-buffered paraformaldehyde for 24 h, and the specimens were paraffin-embedded, sliced at 5 μm, and stained with haematoxylin and eosin. After staining, the sections were rinsed with distilled water, dehydrated, and mounted with Permount. Photomicrographs were taken using an Olympus SZX7 Zoom stereo microscope or BX51 microscope plus Olympus DP71 CCD camera (Olympus Corporation). Software Image-Pro Plus version 6.0 was used to detect the thickness of LV.

Paraffin-embedded slices were also stained with 0.1% picric sirius red (Sigma-Aldrich Inc, St Louis, USA) for fibrillar collagen. Collagen volume fraction (CVF) was expressed as a percentage of the total area of the field occupied by collagen. Analysis of collagen type I and III was performed using a polarized filter. Collagen type I is characterized by a red/yellow and type III by green collagen fibrils.

### Data analysis

All quantitative values are given as mean ± S.E. Mean values of data from different treatment groups were compared using one-way ANOVA. After confirming the equal variances, least-significant difference (LSD) was used to compare the difference between groups. *P *< 0.05 was considered to be statistically significant.

## Results

### SalB binds with MMP-9 according molecular docking and LIGPLOT analysis

**Figure 1:**
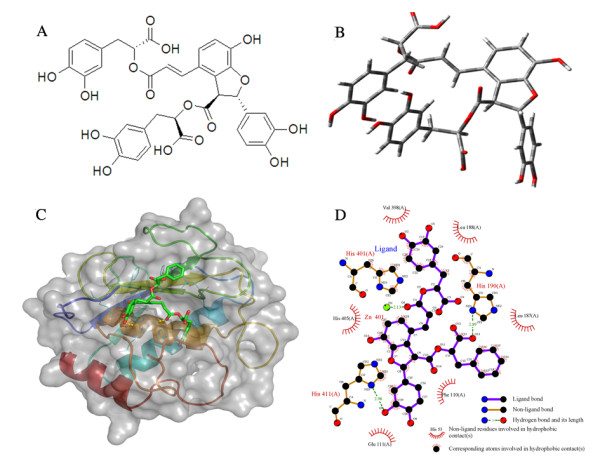
**The molecular docking of SalB with MMP-9**. (**A**) Chemical structure of SalB. (**B**) 3D structure of SalB. (**C**) Docking solution of SalB in the catalytic domain of MMP-9. Protein backbone of MMP-9 (2OVX) is shown in cartoon model, and protein surface is shown in transparency. Selected protein residues interacted with SalB are shown in line model, SalB in stick model, and the yellow dashed lines denote the hydrogen bonds between ligand and the enzyme. (**D**) 2D representation of SalB and MMP-9 interaction was analyzed using LIGPLOT. H-bond is represented as dashed line, and spiked residue represents hydrophobic contacts.

To elucidate the binding of SalB to MMP-9 protein, docking studies were performed to gain insight into the most probable binding conformation. The chemical structure of SalB was shown in Fig. 1A. The 3D structure of SalB was constructed using Corina online demonstration, and was shown in Fig. 1B. Structural coordinate for MMP-9 protein (2OVX) was retrieved from the Protein Data Bank, and AutoDock which can predict the direct binding of proteins with small molecule was adopted to verify the possible docking of SalB to MMP-9. The data suggested that SalB almost occupies the catalytic active sites of MMP-9 completely with a dock score of -10.44 (Fig. 1C).

The LIGPLOT analyses were then introduced to help to understand the in-depth interaction pattern between SalB and the active site residues of MMP-9 protein. Forty-four hydrophobic interaction atom pairs (between carbon atoms of SalB and GLU111, HIS190, HIS401, HIS405, HIS411, LEU187, LEU188, PHE110, VAL398) have been identified using this method. In addition, 2 hydrogen bonds between SalB and MMP-9 protein were detected (Fig. 1D). It is also noteworthy that the distance between SalB O6 (a carbonyl oxygen atom) and zinc is 2.13 Å, suggesting the existence of a coordinated bond between SalB and MMP-9. Because AutoDock does not take coordinated bond formation into account, the bonds between zinc and SalB might be underestimated.

### SalB interacts with MMP-9 at catalytic domain

**Figure 2:**
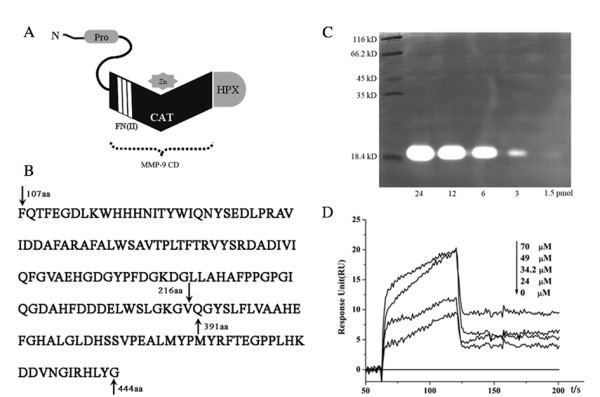
**Direct interaction between SalB and MMP-9 catalytic domain**. (**A**) Representative structural domains of MMP-9. (**B**) Amino acid sequence of recombinant MMP-9 CD. (**C**) MMP-9 CD displayed significant collagenase activity detected by zymography assay. (**D**) Direct binding of SalB to MMP-9 by BIAcore analysis. The binding affinity of SalB with MMP-9 CD was reflected by response unit (RU) values. Immobilization of MMP-9 CD on the Biacore biosensor chip resulted in a resonance signal of 8000 RUs. The kinetic measurements were performed in triplicate using a set of serial dilutions as shown.

MMP-9 has several representative structural domains (Fig. 2A). As described in detail in the method section, we successfully constructed, expressed, and purified the CAT domain that includes three FN(II) inserts (MMP-9 CD) for the binding and enzyme kinetic studies. The sequence of the constructed peptide was shown in Fig. 2B. The recombinant MMP-9 CD displayed significant collagenase activity using in-gel zymography assay, and its' activity on degradation of substrate was dose dependent (Fig. 2C).

The bioreactive MMP-9 CD protein was then used to verify the direct interaction between SalB and MMP-9, employing plasmon resonance by BIAcore. Binding kinetics of SalB and MMP-9 were found to be in a dose dependent manner (Fig. 2D). The equilibrium dissociation constant (K_D_) for SalB was determined using steady state affinity fit and calculated to be 39.5 μM. Taken together, our data provided evidence for the direct binding of SalB to MMP-9.

### SalB inhibits MMP-9 activity competitively

**Figure 3:**
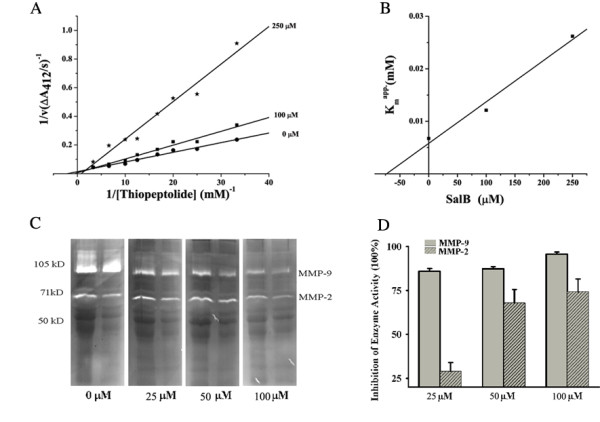
**SalB inhibits MMP-9 activity specifically and competitively**. (**A**) Kinetic analysis of SalB against MMP-9 CD through double reciprocal plots of 1/V versus 1/[thiopeptolide]. (**B**) Secondary plot of K_m_^app ^versus different SalB concentration. The K_i _of SalB against MMP-9 CD is calculated to be 79.2 μM. (**C**) Representative zymography results for SalB inhibition on native MMP-2, MMP-9 activity *in vitro*. (**D**) Quantitative data from Fig. 3C for SalB inhibition on MMP-9, MMP-2 activity. (n = 3 independent experiments).

The double-reciprocal plots were obtained in the presence of various concentrations of thiopeptolide with or without SalB. The mode of SalB inhibition was competitive for MMP-9 CD (Fig. 3A), with the characteristics of un-parallel lines with different 1/v and 1/[thiopeptolide] axis-intercepts and different slopes. The *K*_i _value of SalB on MMP-9 CD was calculated 79.2 μM (Fig. 3B).

To further confirm the inhibition of SalB on native MMP-9, we performed In-gel zymography assay using proteins isolated from MI rat heart because of the abundance of MMP-9 and MMP-2 at 24 h. Briefly, the protein from infarct area was extracted, electrophoresed and incubated in substrate buffer containing indicated concentration of SalB at 37°C overnight. The activities of MMP-9, MMP-2 decreased with the incubation of SalB dose-dependently (Fig. 3C). The inhibition of SalB on MMP-9 activity was 86.0 ± 1.5%, 87.3 ± 1.2% and 95.7 ± 1.2% at 25 μM, 50 μM and 100 μM. And the inhibition of SalB on MMP-2 activity was 29.0 ± 4.9%, 68.0 ± 7.6% and 74.3 ± 7.2% at 25 μM, 50 μM and 100 μM respectively (Fig. 3D), suggesting SalB is more specific on MMP-9 than on MMP-2.

### SalB down-regulates MMP-9 activity instead of protein expression at infarct area of MI rat hearts

**Figure 4:**
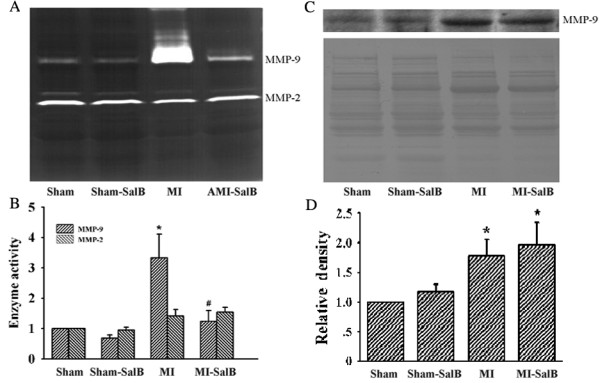
**Selective *in vivo *inhibition of SalB on MMP-9 activity without down-regulating MMP-9 protein expression**. (**A**) The representative zymogram for *in vivo *MMP-9 and MMP-2 enzymatic activities in each treatment group. (**B**) Quantification of data presented in Fig.4B, expressed as fold increase versus Sham. (**C**) Western blot analysis on MMP-9 expression in rats from each treatment group (upper panel). Same amount of protein loaded on each lane was verified by membrane stained with amino black (lower panel). (**D**) Quantification of data presented in Fig. 4C. Expression level of MMP-9 was expressed as fold increase versus Sham. n = 10 for each group. All of the values are expressed as mean ± S.E. *P < 0.05 versus Sham rats; #*P *< 0.05 versus MI rats.

It was reported that up-regulation of MMP-9 was found during the first two weeks after myocardial infarction [15]. To detect the inhibition of SalB on MMP-9 *in vivo*, we evaluated the activity of MMP-9 in infarct heart of rat after 24 h infarction using in-gel gelatin zymography (Fig. 4A). MMP-9 activity was elevated in MI group compared with Sham group (3.33 ± 0.10 fold; *P *< 0.05), and this elevation was inhibited by SalB (Fig. 4B). No significant regulation of SalB on MMP-2 activity was found. To further clarify the regulation of SalB on MMP-9 activity correlated with protein expression or not, we performed Western blotting. Considerable increase of MMP-9 protein was detected in MI group; but treatment with SalB did not alter MMP-9 protein expression (Fig. 4C; upside). Same amount of protein loaded on each lane was verified by the stained membrane using amino black (Fig. 4C; below). The quantification data was shown in Fig. 4D, about 1.79 ± 0.27 fold elevation for MMP-9 expression was found in MI group compared with Sham group (*P *< 0.05).

### SalB prevents the decrease of LV wall thickness

**Figure 5:**
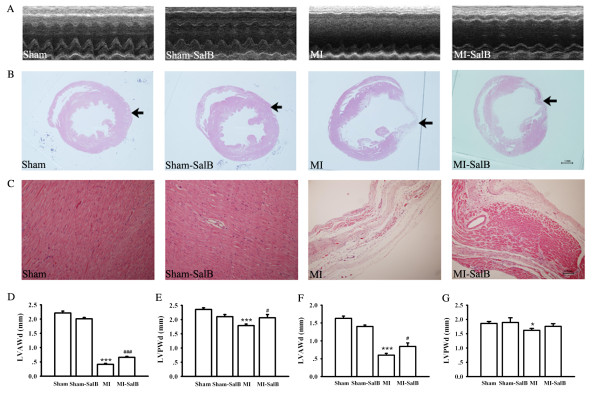
**SalB attenuates LV geometric changes induced by myocardial infarction**. (**A**) Representative echocardiography recordings from each treatment group. n = 20 for every group. (**B**) Representative histopathological patterns of whole heart from different treatment groups. n = 10 for every group. (**C**) Higher magnification (100X) of Fig. 5B for the area indicated by arrows. (**D, E**) Quantitative data of LVAWd (D) or LVPWd (E) determined by echocardiography. (**F, G**) Quantitative data of LVAWd (F) or LVPWd (G) calculated using histopathology. *P < 0.05, ***P < 0.001 versus Sham rats; #*P *< 0.05, ###*P *< 0.001 versus MI rats.

The effect of SalB treatment on LV remodeling was investigated using echocardiography and histopathological evaluation at 8 weeks after MI. The representative echocardiography recordings (Fig. 5A), and histopathological structure (Fig. 5B) of whole heart, from four group of animals are shown. In Sham and Sham-SalB animals, concentric circle form of LV chamber and uniform wall thickness were quite evident (Fig.5B). However, significant dilatation of LV chamber was found in MI group. Dilatation of asymmetric chamber in MI-SalB group was reduced compared that with MI alone, suggesting SalB may provide protective benefit against LV remodeling. At a higher magnification, transmural myocardial infarction and aneurysm were readily detected in MI group at the position indicated by arrow, but were absent in the other groups (Fig. 5C). Both LV anterior wall end-diastolic thickness (LVAWd) and LV posterior wall end-diastolic thickness (LVPWd) were decreased in MI group compared with that of control when quantified using echocardiography (Fig. 5D and 5E) or histophathology (Fig. 5F and 5G) measurements. Such decrease in wall thickness was alleviated considerably in MI-SalB group.

### SalB improves left ventricle contractility

**Figure 6:**
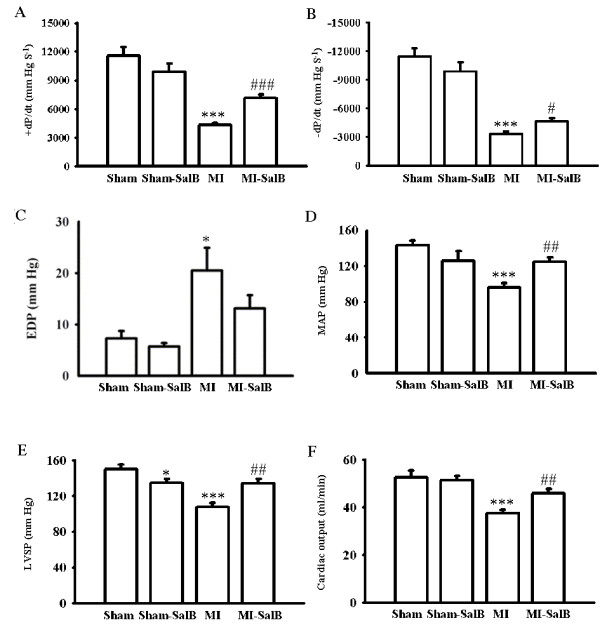
**Effects of SalB treatment on hemodynamic parameters**. Maximum rate of pressure development (+dP/dt_max_), maximum rate of relaxation (-dP/dt_min_), end-diastolic pressure (EDP), mean arterial pressure (MAP), Left ventricular systolic pressure (LVSP) and cardiac output were demonstrated. All the values are expressed as mean ± S.E. *P < 0.05, ***P < 0.001 versus Sham rats; #*P *< 0.05, ##*P *< 0.01, ###*P *< 0.001 versus MI rats. n = 20 for every group.

The major haemodynamic parameters were measured to evaluate the left ventricle contractility (Fig. 6). The left ventricle dysfunction in the MI rats was confirmed with a significant decrease of +dP/dt_max _(4320.7 ± 198.9 mmHgS^-1 ^versus 11565.4 ± 942.3 mmHgS^-1^, *P *< 0.001), -dP/dt_min _(-3310.0 ± 288.6 mmHgS^-1 ^versus -11478.8 ± 819.5 mmHgS^-1^, *P *< 0.001), MAP (96.1 ± 4.9 mmHg versus 143.1 ± 5.6 mmHg, *P *< 0.001), LVSP (107.6 ± 5.0 mmHg versus 149.9 ± 5.4 mmHg, *P *< 0.001), and increase of EDP (20.5 ± 4.4 mmHg versus 6.9 ± 1.7 mmHg, *P *< 0.05) compared to the Sham group. SalB treatment partially reversed the impairment of left ventricle function by improving values of measured parameters; +dP/dt_max _(7151.1 ± 362.8 mmHgS^-1 ^versus 4320.7 ± 198.8 mmHgS^-1^, *P *< 0.001), -dP/dt_min _(-4634.2 ± 357.0 mmHgS^-1 ^versus -3310.0 ± 288.6 mmHgS^-1^, *P *< 0.05), MAP (124.8 ± 4.9 mmHg versus 96.1 ± 4.9 mmHg, *P *< 0.01), LVSP (134.4 ± 4.8 mmHg versus 107.6 ± 5.0 mmHg, *P *< 0.01) compared with MI group.

To further evaluate the cardiac function of rats in different groups, transonic flowmetery was used. Compared with Sham group, MI rats had a decrease in value of cardiac output from 52.7 ± 2.7 ml/min to 37.5 ± 1.6 ml/min (*P *< 0.001). In MI-SalB group, the cardiac output reversed to 45.9 ± 1.9 ml/min (*P *< 0.01).

### SalB prevents myocardiac fibrosis induced by MI

**Figure 7:**
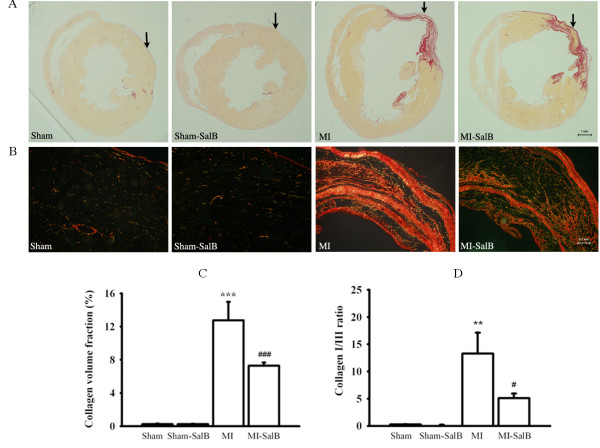
**SalB decreases fibrosis induced by MI**. (**A**) Representative pictures of whole heart stained by Sirius red. The position of collagen deposition was stained in red.(**B**) Higher magnification (100X) of areas in Fig. 7A indicated by arrows using a polarized filter. The collagen I was demonstrated as red/yellow and collagen III was demonstrated as green. (**C**) Quantitative data of collagen volume fraction. (**D**) Quantitative data of the ratio of collagen I/III in infarct area. **P < 0.01, ***P < 0.001 versus Sham rats; #*P *< 0.05, ###*P *< 0.001 versus MI rats. n = 10 for every group.

It is known that alteration of collagen deposition and collagen type may contribute to congestive heart failure, likely involving MMPs. We investigated the effects of SalB on myocardiac fibrosis to further our understanding of the mechanism of improved cardiac performance by SalB treatment. Fig. 7A showed the representative pictures of whole heart stained by Sirius red. It was known that development of congestive heart failure is associated with the increase in the ratio of collagen type I/III. Fig. 7B showed the magnification of Fig. 7A at the position indicated by arrows using a polarized filter, through which the collagen I was demonstrated as red/yellow and collagen III was demonstrated as green. SalB markedly attenuated an increase in collagen volume fraction in rats with MI, and the quantitative data was shown in Fig. 7C (7.28 ± 0.39% versus 12.8 ± 2.21%; *P *< 0.001). Fig. 7D showed that SalB not only reduced total fibrosis but also decreased the ratio of collagen I/III in infarct area (5.10 ± 0.86 versus 13.3 ± 3.8; *P *< 0.05).

### The main metabolites of SalB do not inhibit and interact with MMP-9 CD

**Figure 8:**
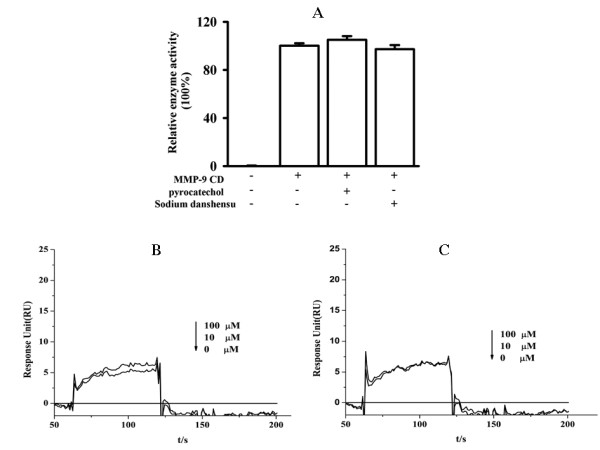
**Main SalB metabolites do not bind MMP-9 CD nor inhibit its activity**. (**A**) No inhibition was found for the two main metabolites of SalB, pyrocatechol and sodium danshensu, on MMP-9 CD activity. (**B, C**) Biacore analysis for binding of compound with MMP-9. No interaction was found for pyrocatechol (B) or sodium danshensu (C).

Our previous work showed that pyrocatechol and danshensu were two main metabolites of SalB with oral dosing in rat [16]. In this study, we examined the potential effect of these two SalB metabolites on MMP-9 activity using spectrophotometric enzyme and surface plasmon resonance assays. No inhibition on MMP-9 activity was detected when pyrocatechol or sodium danshensu at saturation concentration of 100 μM for each compound was incubated with 50 nM of MMP-9 CD (Fig. 8A). Similarly, no significant binding of pyrocatechol (Fig. 8B) or sodium danshensu (Fig. 8C) to MMP-9 CD was observed. Our data suggests that it is unlikely that the observed *in vivo *inhibitory effect of SalB on MMP-9 comes from the two metabolites.

## Discussion

MMPs, especially MMP-9, were considered to be one of the major causes leading to extracellular matrix remodeling [17]. Thus, there is growing interest in MMP-9 as a novel therapeutic target for the prevention of LV remodeling. Here, we presented evidence demonstrating for the first time that SalB bound to MMP-9 at the catalytic domain, resulting in inhibition of MMP-9 activity *in vitro *and *in vivo*. SalB did not exhibit significant effect on MMP-2 activity and its inhibition on MMP-9 was unlikely derived from its main metabolites, pyrocatechol and danshensu. Along with its regulation on MMP-9 activity *in vivo*, SalB treatment also led to a less impaired LV chamber geometry, attenuated myocardial fibrosis, and thus improved heart performance of MI rats.

MMP-2 and MMP-9 have demonstrated substrate affinities for denatured fibrillar collagen, collagen type IV, fibronection, laminin, elastin and proteoglycans [18]. The structures of MMP-2 and MMP-9 are highly similar; however, the three-dimensional structures of MMP-2 or MMP-9 are unique among MMPs. The catalytic domain of MMP-2 and MMP-9 carries an insert of three fibronection type-II-like modules that mediates interaction with the substrates [19]. The main differences between these two proteins are in the S1' substrate or selectivity pocket [20]. Kiyama et al. reported that MMP-9 holds a pocket-like S1' subsite with a floorboard and MMP-2 has a channel-like S1' subsite [21]; however, based on the results of docking analysis, the different structure at S1' subsite did not contribute to the selectivity of SalB towards MMP-9 than MMP-2 because the binding sites of SalB on MMP-9 did not locate at this domain. SalB showed a higher dock score for MMP-9 than MMP-2, of -10.44 and -8.84 (data not shown) respectively, suggesting that the higher Van der Waals force between SalB and MMP-9 active sites contributes to the selectivity.

Due to the prominent role of MMPs in physiological and pathological processes, large endeavors were put to develop pharmacological MMP inhibitors [22]. Several MMP-9 inhibitors have been reported including AM-409 (phosphinic acid), RO-206-0222 (pyrimidine-2, 4, 6-trione), An-1 and MJ-24 (carboxylat), and MS-560 (trifluoromethyl hydroxamic acid inhibitor). Comparing with these compounds, the docking sites of SalB on MMP-9 are very similar. Almost all of these compounds interact with MMP-9 at the bulge-edge segment (Gly186, Leu188, Ala189) and the S1' wall (Leu418, Tyr420, Pro421, Tyr 423, Arg424). It is also a well known fact that most synthetic MMP inhibitors contain a chelating group (hydroxamic acid, carboxylate or thiol group) for zinc ligation and a peptidic or peptidomimetic moiety that used the same substrate binding sites [23]. Strikingly, SalB has a unique structure compared to others listed above.

A growing body of literature supports a major role of MMP inhibitor-PD166793 (C_17_H_18_BrNO_4_S) to counter the maladaptive LV remodeling process in multiple animal models of congestive heart failure [24]. However, major obstacles which prevent PD166793 and other MMP inhibitors from further development are the serious side effects [25]. *Salviae miltiorrhizae *has been used in China for many years to treat various diseases including heart failure with little side effects [26]. As the main component of *Salviae miltiorrhizae*, SalB is considered to be a candidate as a single agent for clinic use. Unlike PD166793 which has a broad spectrum of MMP targets, we showed preliminary data suggesting that SalB is probably a more selective towards MMP-9 compared to other MMPs. Pharmacological effect of SalB was demonstrated in MI rats in our study for improving structure and function of impaired MI heart with no obvious toxicity. It is quite hopeful that the unique structure of SalB, and its relative selectivity towards MMP-9, will enable us to explore the structure-activity relationship to identify more specific and safer MMP-9 inhibitors for the treatment of cardiovascular disease.

## Conclusions

Improvement of heart function after MI is directly associated with inhibition of MMP-9 activity. In the current work, we have provided in great detail of biochemical analysis on SalB structure and its selective, competitive inhibition on MMP-9 activity. We also examined potential downstream mechanism of SalB in regulating heart geometry and function in MI rat model. The unique structure of SalB and its specificity towards MMP-9 are useful information for developing a new lead compound of MMP-9 inhibitors to be test in clinic.

## List of abbreviations

MI: myocardial infarction; LV: left ventricular; SalB: salvianolic acid B; MMP-9: matrix metalloproteinase 9; ECM: extracellular matrix; LVAWd: LV anterior wall end-diastolic thickness; LVPWd: LV posterior wall end-diastolic thickness; +dP/dtmax: maximum rate of pressure development; -dP/dtmin: maximum rate of relaxation; EDP: end-diastolic pressure; MAP: mean arterial pressure; LVSP: left ventricular systolic pressure

## Authors' contributions

BJ, LX and YD performed animal experiments. YW, JC and FX performed enzyme detection. ZG performed docking and molecular modeling computations. XS and DG designed, analyzed experiments and prepared the manuscript. All authors read and approved the final manuscript.
